# Eighty-six billion and counting: do we know the number of neurons in the human brain?

**DOI:** 10.1093/brain/awae390

**Published:** 2024-11-27

**Authors:** Alain Goriely

**Affiliations:** Mathematical Institute, Andrew Wiles Building, University of Oxford, Radcliffe Observatory Quarter, Oxford, OX2 6GG UK

## Abstract

Through statistical analysis and comparison of different studies, Alain Goriely challenges the widely accepted figure of 86 billion neurons in the human brain, and argues that the actual number is uncertain, with estimates ranging between 61 and 99 billion.

***The current estimates for the number of neurons in the human brain cannot be properly justified.***

## Introduction

When the number of entities in a system is large, it becomes impossible to enumerate them all, and indirect techniques for counting are needed. We will never know exactly how many stars are in the Milky Way or how many humans have been alive in the history of the world. In these cases, statistical methods based on clear assumptions can be used to provide estimates with a given range. Typically, we rely on the notion of density. If we know how many red blood cells there are on average in a given volume of blood (around 5 trillion per litre), and we know on average the total volume of blood (around 5 l), we can obtain a reasonable estimate of the number of red blood cells in humans (around 25 trillion).

Knowing how many things there are in a system is central to most scientific disciplines. In physics, it is the first step in characterizing matter. In chemistry, the fundamental definition of a mole (exactly 6.02214076 × 10^23^ units) expresses the importance of counting the number of entities in a given weight. In engineering, one expects that any piece that belongs to a device is catalogued before assembly so that, in principle, one can know the number of parts.

When it comes to the brain, the situation is not that simple due to the heterogenous nature of brain tissues. Neuroscientists will study the most intimate details of cells and proteins and gather immense datasets about every possible measurable aspect of the brain. Yet, a naive query such as how many neurons are in the human brain is not a central question for the field. A cursory look at the literature shows that, in recent times, there has been an almost universal agreement that there are 86 billion (bn) neurons in the brain. Indeed, many scientific papers on the brain, including mine, start with a statement in the form: ‘the human brain contains 86 billion neurons’, followed by a citation to one of the most cited papers in neuroscience from the group led by Prof. Herculano-Houzel,^1^ who has worked extensively on challenging neuromyths and was the first to address the question truly.

In the same spirit that drove her initial study, the goal here is to challenge the accepted fact that the human brain contains 86 bn neurons. Indeed, I will show that the data from the original and subsequent studies on neuron numbers do not warrant this generic conclusion and that the only statements that can be made about the number of neurons are much weaker.

## The situation pre-86 billion

In an excellent review of the topic, Prof. Herculano-Houzel and colleagues provide a remarkable account of the history of the problem.^2^ Before their ground-breaking work in 2009, the accepted number of neurons varied widely from the first estimate in 1895 of 3 bn to a puzzling 1 trillion in 1981, until it settled mysteriously to 100 bn around 1991. Until 2009, 100 bn was the accepted estimate and is still widely quoted. The number 100 is what we call a round number, and its roundness suggests that it is a rough estimate and not a measured quantity obtained through scientific study. The absence of a clear reference or study associated with this number further indicates that the problem was not settled with any scientific rigour. It just emerged in the literature like mushrooms after a heavy rain.

The landscape changed completely in 2009 with the use of the so-called ‘isotropic fractionator’ to measure cell numbers.^1,3,4^ I will not review or challenge the method itself but constrain myself to an analysis of the data provided by these studies. Yet, it is important to note that neuronal nuclei (NeuN), the protein used as a neuronal marker, is not universally expressed in all neuronal types, and its levels vary with neuronal maturation, increasing postnatally, complicating its use as a marker for neurogenesis.

## The problem with the 2009 paper

Before we proceed, let us look at the data and main claim of the 2009 paper.^1^ Following the text, we know that ‘brains from 50-, 51-, 54-, and 71- year-old males, deceased from nonneurological causes and without cognitive impairment (CDR 0, IQCODE 3.0), were analyzed’. The average weight of the four brains is 1508.91 g, with a standard deviation of 299.14 g. The weight of each brain or the number of neurons is not given. However, according to the paper, the average number of neurons in the four brains is 86.06 bn, with a standard deviation of 8.12 bn. We are further provided with a range of 78.82 to 95.40 bn. We can then combine these data to reconstitute the original dataset and find that the numbers of neurons are: {78.82, 79.72, 90.30, 95.40} bn. From this small dataset, the authors make the most important claim that: ‘We find that the adult male human brain contains on average 86.1 ± 8.1 billion NeuN-positive cells (“neurons”)’. With over 3000 citations, it is probably the single most cited number about the brain.

It is quite an achievement, and the low number of data-points in the study demonstrates how difficult the process must have been. But before we close the matter, let us reflect on what can really be concluded from four data-points, as we know that statistical statements depend strongly on the number of data points. If we have one or two data-points, we would gladly agree that a mean would not be representative of the entire population. How about four?

Statisticians have studied this type of problem in great detail: if we sample *n* ≥ 2 points from a distribution, it is easy to take the sample mean (*µ*) and standard deviation (*δ*) of *n* samples. We want to know to what extent these values are representative of the actual (but unknown) population mean that we are interested in. In our case, the question is: to what extent is *µ* = 86.1 bn with *n* = 4 a valid estimate of the number of neurons in the human brain?

This type of problem is addressed by the so-called ‘standard error of the mean’. Assuming that the underlying population is normally distributed, we can calculate the upper and lower 95% confidence limits (*x*_±_) for the true population mean as:


(1) $$x_{\pm }\;=\;\mu \;\pm \;t^{*}\;\times \;\mathrm{SEM}$$

where *µ* is the sample mean, $\mathrm{SEM}\;=\;\delta /\sqrt{n}$ is the standard error of the mean, with *δ* as the standard deviation of the sample, and t* is the critical *t*-value given by a *t*-distribution. Since it is a two-tailed test (we are considering both tails of the distribution), we need the *t*-value where the cumulative probability is (1 + 1.095)/2 = 0.975 with degrees of freedom *d* = *n*−1 = 3. The critical *t*-value for *d* = 3 at 95% confidence is approximately t* = 3.182.

In our case, we have *µ* = 86.1 bn, *δ* =  8.1 bn, which leads to a margin of error (ME) = t* × SEM = 13.0 bn and to the confidence interval for the population mean (*x̄*) given by:


(2) $$73.1\;\mathrm{bn}\;<\;\bar{x}\;<\;99.0\;\mathrm{bn}$$

Hence, from the data given, the only statement about the mean we can make is that an estimate of the average number of neurons in this study is between about 73 and 99 bn neurons (note that since the estimate is so vague, I see no point in providing it with decimal fractions of a billion). This is a very different statement than giving 86 bn as an average. Unfortunately, we cannot make any other statement since the data from this study are not public and repeated requests to the corresponding author for clarification and data remain unanswered.

A difference of 26 bn is rather large, but by itself it could be considered a technical correction made by a pedantic mathematician. Yet, the problem becomes more acute when we look at another study that uses the same method.

## Another study, another number

The only other study on the total number of neurons in the human brain that I was able to find was performed by Roberto Lent’s group using the same technique and published 4 years later.^5^ The data are from five elderly females with no cognitive impairment between the ages of 71 and 84, who died of non-neurological causes. I am grateful to Professor Lent for responding to my queries and for sharing their data. The dataset with *n* = 5 is {62.1, 67.3, 63.3, 72, 72.0627} bn. We can now repeat the same computation with *µ* = 67.3 bn, *δ* = 4.6 bn, *d* = 4 and t* = 2.776 to obtain:


(3) $$61.5\;\mathrm{bn}\;<\;\bar{x}\;<\;73.1\;\mathrm{bn}$$

**Figure 1:**
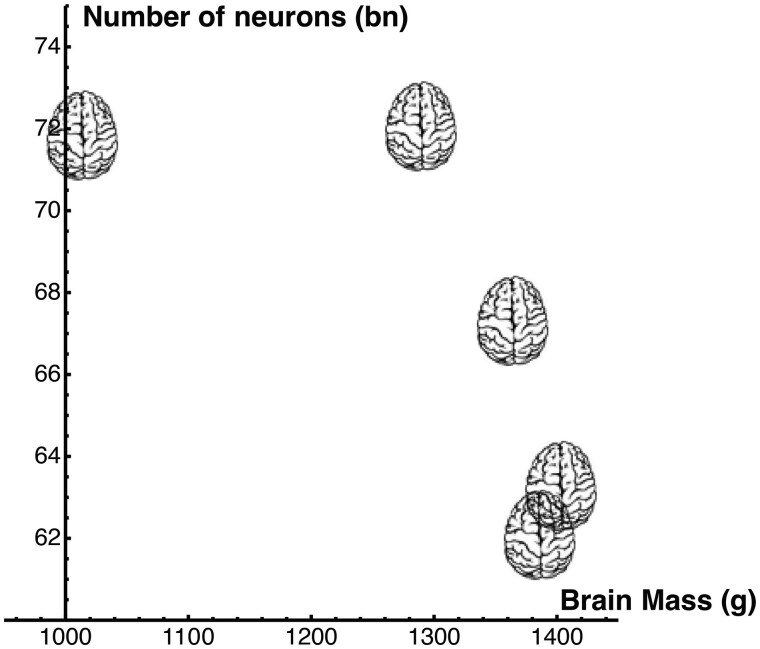
**Number of neurons versus brain mass.** For the available data from *n* = 5 female brains, the number of neurons versus brain mass does not show any clear pattern.

The data also include the brain masses {1385.93, 1363.9441, 1403.24, 1013.70, 1289.32} g, with no clear relation to the number of neurons, as shown in Fig. 1 (the second lightest brain has the highest number of neurons and the second heaviest brain has the lowest number of neurons).

## Comparing the two datasets

Despite the fact that both groups claim to follow the same procedure, there is no clear explanation for the differences between the two studies, and we are left to speculate:

- The tentative *post hoc* explanation in 2015 for the difference between the two sample means in the two studies, namely that ‘biological variation may account for 25% or more, especially in humans and with small sample sizes’,^6^ is not satisfactory since there is no rationale for a variation of 25%, and if this type of variation occurs, it should have been observed in each of the two experiments.
- There is a large difference in average brain weights (1291 g versus 1509 g). If we assume a purely proportional effect, it would boost the sample mean from 67.3 bn to 67.3 × 1509/1291 = 78.6 bn, which would bring the two datasets much closer. However, as discussed earlier, no clear trend emerges between volume and neuron numbers in the second dataset (data not available for the first dataset).
- The difference in sex between the two populations may not be an explanation either, as there is no evidence of systematic differences between the numbers of neurons in male and female brains of the same weight.
- There is a marked difference in ages between the two groups. Change in brain volume with ageing is well established,^7,8^ but the extent to which it would contribute to a large change in the number of neurons is unknown. Indeed, the relationship between brain volume and neuron number has not been established, and it is now believed that there is very little decline in cortical neuron numbers during normal ageing.^9^
- Another possibility is that, within a given experimental set-up, systematic errors are being made and not identified. It is clear that the landmarks for dissections, dehydration, post-mortem time, age and sex can vary. These errors may differ among experiments, leading to different estimates.^4,10^ In short, we have no real information about the variability of the method itself with respect to multiple factors.

## Statements

We have reached a difficult point: the two estimates are widely different, with no overlap and no clear explanation, which does not give us any confidence in a consensus. The only statements that we can make are much weaker:

- Experiments have shown variations between 62 and 94 bn neurons in the human brain (*n* = 9).
- An experimental study on the number of neurons suggests an average between 73 and 99 bn neurons in the healthy male human brain (*n* = 4).
- An experimental study on the number of neurons suggests an average between 61 and 73 bn neurons in the healthy female human brain (*n* = 5).

Clearly, none of these statements is satisfactory or as catchy as ‘the human brain has 86 billion neurons’. Yet, they are the true reflection of our knowledge. We cannot present a more precise assertion without more available data.

It is worth noting that the problem is not unique to neuroscience: astrophysicists have faced similar challenges when trying to establish the number of stars in our galaxy, with the same lack of success, which has gone from an accepted 100 bn stars to newer estimates of 200 to 400 bn.

## Does it matter?

No and Yes. In the case of red blood cells, it is of the utmost importance to know the red blood cell count (the number of cells per unit volume), as this is a critical biomarker for anaemia, polycythaemia and other conditions. Therefore, the variability in blood cell concentration has been studied extensively and is well understood. However, in the case of the brain, there is no medical need to know either the total number or the concentration of cells in its different parts. Therefore, the question is not as important, and when neuroscientists hear or write the number 86 bn, they are most likely fully aware that it is an estimate that comes with certain assumptions and uncertainties. It should also be clear that most papers that cite this number in an introduction never actually use its value subsequently. Such numbers serve mostly a comparative purpose, either to study how different parts of the brain contribute to the total number of neurons or to compare different species (which also suffer from the same sampling problem). In such cases, we can hope that the same bias and distributions affect other similar numbers so as to not affect the overall conclusions of the studies. We could argue that very little damage is done by assigning a number to the brain to satisfy our basic human curiosity about ‘how many things are there in this thing?’.

Yet scientists are not children who need to be appeased, and numbers should not be used as pacifiers. We should strive for rigour and insist on precision in all instances, even when scientific facts are awkward to formulate or their mis-formulation does not have dire consequences. Why claim that the brain has 86.1 bn neurons if the data do not support it? Whether or not the number is just used in an introduction or implicitly assumed to be an estimate, when we state it, we make it a scientific truth and bias any further investigation. More dangerously, it has been used to establish scaling laws of neuron numbers with respect to body weight, which are then used in other contexts, forever stacking uneven blocks on a scientific tower built on sand.

Finally, I would like to reiterate that my comments here in no way diminish the accomplishments of the different groups involved. They started with a difficult question that had previously been ignored and found an experimental way to obtain estimates for the number of neurons. This is a remarkable achievement that has been used to challenge established ideas and open new avenues of research. Yet, from the data available, the only possible answer to the titular question ‘Do we know the number of neurons in the human brain?’ is: No, we don’t.
